# Radiotherapy in the Adjuvant and Advanced Setting of CSCC

**DOI:** 10.5826/dpc.11S2a168S

**Published:** 2021-10-01

**Authors:** Paolo Muto, Francesco Pastore

**Affiliations:** 1 Radiation Oncology Unit, Istituto Nazionale Tumori - IRCCS Fondazione G. Pascale – Naples, Italy; 2 Radiation Oncology, Fondazione Muto Onlus - Emicenter – Naples, Italy

**Keywords:** radiotherapy, squamous cell carcinoma, brachytherapy

## Abstract

**Introduction:**

The use of radiotherapy for cutaneous squamous cell carcinoma (CSCC) has solid historical roots. It is used with patients who are not suitable for surgery, with patients with high-risk histological features in the adjuvant setting, and in palliative care.

**Objectives:**

The aim of this article is to summarize and provide a radiation therapy overview on the indications, effectiveness, and potential adverse events of radiotherapy in the adjuvant and advanced setting of CSCC.

**Methods:**

We performed a comprehensive literature review on PubMed, adopted as our biomedical literature database. Articles were selected based on their date of publication (in the last 30 years) and relevance.

**Results:**

Radiotherapy (RT) can safely be used to manage non-surgical patients and high-risk patients in the advanced CSCC setting. The remarkable progress of delivery techniques has greatly improved the effectiveness and toxicity profile of RT treatments. From 2D techniques to intensity modulated radiation therapy (IMRT), and brachytherapy, all RT techniques have greatly advanced. To improve acute and chronic side effects, a deeper care has been used. As regards CSCC, several dose fractionations and schedules have been suggested, in line with the patient’s age and medical conditions.

**Conclusions:**

RT is a fundamental and constantly evolving therapeutic option in the treatment of CSCC, to minimize the risk of recurrence and metastases in the adjuvant setting and in the exclusive treatment for non-surgical patients. Patients’ selection is crucial, together with and a collaborative team working approach among the specialists involved in disease management in the perspective of the best multidisciplinary assessment.

## Introduction

Cutaneous squamous cell carcinoma (CSCC) is the second most common type of non-melanoma skin cancer (NMSC) after basal cell carcinoma (CBCC). It arises most commonly in sun-exposed areas of the body and originates from keratinocytes in the epidermis through a carcinogenesis process [1]. As the world’s population ages, the incidence of both types of NMSC (SCC and BCC) is dramatically increasing and disease implications on public health are vastly underestimated in terms of morbidity and treatment costs. These costs are unavoidably going to rise [2]. CSCC can arise from precancerous precursor lesions such as actinic keratosis or can grow de novo, in particular on chronically inflamed skin and consequently more exposed to pro-carcinogenetic stimuli [3].

Invasive CSCC (histologically characterized by the presence of infiltrating cells crossing the basement membrane) has the ability to relapse and metastasize to regional lymph nodes or distant organs and, if left untreated or if inadequately treated, can lead to extensive tissue destruction up to massive patterns of tumour infiltration. Even though the ability of CSCC to metastasize is limited, the presence of distant metastases in CSCC patients is associated with poor prognosis and a median survival of less than 2 years. For this reason, it is fundamental to perform a careful clinical and surgical evaluation, that goes hand in hand with a correct early management of all cases. Multidisciplinary management is essential in order to guide the patient towards the best possible treatment options [4]. The ultimate SCC treatment goals are complete removal of the tumor or, when this is not possible, tumor debulking, and the minimization of functional and aesthetic impairment that often, for particular anatomical areas, can be a very central issue. [1]. Surgery with adequate margins is the most common treatment option for most CSCCs, but radiation therapy (RT) can be an effective non-surgical option in the definitive (inoperable patients), adjuvant (high postoperative risk), and palliative (cytoreductive, pain-relieving) setting. RT is also a tissue-preserving modality that might offer a better cosmetic and functional outcome than surgery [4].

A variety of radiation therapy techniques have been used to treat epithelial skin tumors. NMSC have historically been among the first to be irradiated since discovery of radioactivity. The appropriate radiation therapy technique depends on multiple factors, including the primary tumor’s location, the neoplasm size, the scar length (in the adjuvant setting) which may be difficult to cover with radiation planning, the surrounding anatomy (presence of organs at risk that must be preserved), and the presence of disease-affected lymph nodes that deserve to be included in the radiation field [5]. Surface electrons are typically used for primary lesions or tumor beds > 5 mm or deeper, because of their unique physical penetration features. Electrons are produced by modern linear accelerators and have a dose distribution with a peak dose near the skin surface and a rapid dose drop beyond the target. This allows adequate coverage of the disease or surgical bed and the ability to minimize side effects. Superficial X-rays, or photons, have greater physical penetration compared with surface electrons and have been used to treat deep CSCC or more advanced diseases (with or without positive nodes) [6].

This article aims to summarize the effectiveness, potential adverse events, and indications of RT in the adjuvant, advanced, and palliative setting of CSCC, performed through a thorough and broad literature review.

### When is Radiotherapy Recommended?

RT is recommended as adjuvant therapy in high-risk CSCC or when surgery is excluded due to cosmetic or functional reasons [6]. RT allows treatment of anatomic sites that are difficult to manage surgically, and to achieve a good cosmetic result. This applies in particular to the head and neck (H/N) region. RT is also preferred for elderly patients (> 60 years) [7]. The dose of RT can be delivered with external beams (external beam radiation therapy (EBRT)) and by direct application using brachytherapy (BRT). EBRT is delivered via photon or electron beams and can be superficial or deeply penetrating, depending on the megavoltage of the different energy sources [8]. RT can be delivered to fields of different sizes and with different complex shapes. Highly conformal RT, such as tomotherapy or volumetric modulated arc therapy (VMAT), allows delivery of relatively superficial RT to complex and often irregular targets, while limiting the dose to adjacent organs at risk (OARs) [9, 10]. Radiation therapy can be administered via different techniques, several fractionations, and total doses. The aim is to match the tumor and spare healthy tissues from radiation. RT delivery has greatly improved, starting from a better visualization of the target with ever-improving imaging techniques, through precise contouring and treatment planning systems. Many quality-control checks have been added in the intra- and inter-fraction assurance. The choice of the technique is certainly influenced by the type of tumor, treatment setting (radical, adjuvant, or palliative), tumor depth, and location of the tumour that can be particularly unfavorable and close to sensitive organs at risk. High-energy radiation therapy, delivered by a linear accelerator, has greater penetration capacity and is therefore useful to treat deeper malignant tumors while largely sparing the skin. Low-energy radiation (kilovoltage and orthovoltage) is preferred to treat skin lesions where deep penetration is not necessary and skin preservation is the main concern [11, 12].

RT is usually a well-tolerated treatment with specific acute and late toxicities and documented advantages and disadvantages compared with surgery. The most striking limits of radiation therapy are adverse effects and contraindications. Skin reactions triggered by radiation therapy are called radiation therapy-induced dermatitis or radiodermatitis. They can be acute (up to 6 months after the end of treatment) or delayed. They are related to the dose delivered and the anatomical location. Acute reactions lasts several weeks, patients may experience skin changes (ranging from faint erythema and desquamation to skin necrosis) and ulceration, depending on the severity of the reaction. Delayed reactions usually appear months or years after treatment and are more common with higher treatment doses. The most common late reactions are hypopigmentation and hyperpigmentation, telangiectasia, epidermal atrophy, skin fragility, sebaceous gland atrophy, alopecia, fibrosis, necrosis, and an increased risk of certain cancers, such as angiosarcoma. RT contraindications include young age (for CSCC is a minor concern), verrucous SCC, cancer-predisposing genodermatoses, and immunodepression (essential cost-benefits analysis) [13].

### Radiotherapy in the Adjuvant Setting

The goal of adjuvant radiation therapy is to reduce the risk of local or regional recurrence after surgical excision. In general, adjuvant radiation therapy is offered when the risk of recurrence is high or the likelihood of successful salvage surgery is relatively low [11]. Risk factors to look out for in CSCC include male sex, recurrent disease, neoplasms located at the center of the face, poor histologic differentiation, and deep subclinical extension. Other high-risk factors for CSCC include tumor location (lip or ear), tumors arising from scarring tissues, size > 2 cm, depth > 4 mm or Clark level ≥ IV, invasion beyond subcutaneous tissues, rapidly growing lesions, perineural invasion, desmoplasia, poor differentiation, and infiltrative margins [14]. Adjuvant radiation therapy is recommended after extensive surgical excision with close or positive margins (only if the tumor cannot be re-excised) or in the presence of high-risk factors, including perineural invasion (PNI), invasion of bone or nerves, or in case of recurrent disease after previous surgical excision or other medical therapy [15]. The rate of positive margins after excision in CSCC ranges from 5.8% to 17.6% and is influenced by the adopted technique [16,17]. Positive margins after surgery have been reported as prognostic in a study [18]. Indeed, CSCCs with a close or positive surgical margin have an increased risk of local recurrence and locoregional metastasis [19]. Postoperative RT is a treatment option for tumors with margins that are not completely excised or cannot be completely resected. In a study of CSCC of the lower lip that included tumors with a close or positive margin, the local recurrence rate was 64% for tumors with positive margins that were not subsequently excised versus a 6% rate for those treated with postoperative RT [20]. PNI is an important risk factor because it has been shown to be associated with a higher risk of recurrence and a higher incidence of lymph node metastasis [18]. PNI occurs in between 2.5% and 14% of CSCCs, usually found as an incidental histologic finding. It has been linked to poor prognosis and a higher rate of metastasis and disease-specific death [14,21,22]. Extensive PNI is an indication for postoperative RT according to NCCN guidelines [23]. Postoperative RT has also been recommended for high-risk tumors.

Historically, the definition of high risk has been a matter of debate. Guidelines agree on the definition of high risk as a disease characterized by diameter > 2 cm, a thickness > 2 mm (and especially 6 mm), poor differentiation, ear or lip location, PNI, recurrence, and immunosuppression [24]. Most of the evidence regarding the role of postoperative RT in the other risk is derived from a 2009 systematic review indicating that prognosis is generally excellent as long as margins are negative and PNI is not observed, and therefore postoperative RT is not necessary if no such findings occur [25]. CSCC with cranial nerve invasion, on the other hand, represents a possible criterion for choosing to perform postoperative RT [26]. Several RT schedules have been used for adjuvant treatment of CSCC. Briefly, the proposed algorithm by the NCCN includes doses of 60–64 Gy over 6 to 7 weeks or 50 Gy over 4 weeks [23].

### Radiotherapy in the Definitive Setting

Definitive primary RT represents a curative and alternative treatment strategy to surgery for CSCC. RT may be considered as primary treatment in patients who are not candidates for surgery (eg locally advanced CSCC, comorbidities, or refusal of surgery) or in cases where surgery is not feasible. This may occur when the surgical approach could result in poor functional outcomes or be disfiguring, as in large CSCC lesions located on the face (eg eyelids, nose, and lips) or large lesions on the ear, forehead, or scalp [13]. No prospective randomized trials comparing the efficacy of primary RT in local tumor control and patient survival compared with other local therapy modalities are available. A mean local recurrence rate of 6.4% was reported in a meta-analysis analyzing 1018 CSSCs, including 14 observational RT studies [1]. Also in the exclusive setting, RT dose can be delivered as either EBRT or BRT. EBRT can use both photons and electrons, with deeply penetrating energies in the range of 4–10 MV. Treatment can be administered to a small surface area (eg the nasal wing) or a large complex volume (eg the entire scalp or base of the skull). The total prescribed dose and fractionation should reflect differences in radiobiologic efficacy between radiation modalities. Briefly, doses of 45–50 Gy in fractions of 2.5–3 Gy are recommended for tumors < 2 cm and doses of 60–66 Gy in fractions of 2 Gy or 50–60 Gy in fractions of 2.5 Gy for tumors > 2 cm [1].

**Figure 1:**
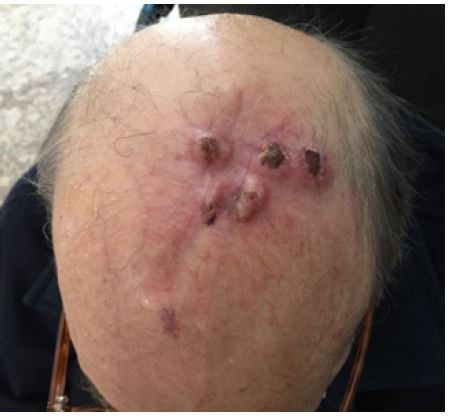
Pre RT treatment for a CSCC.

**Figure 2:**
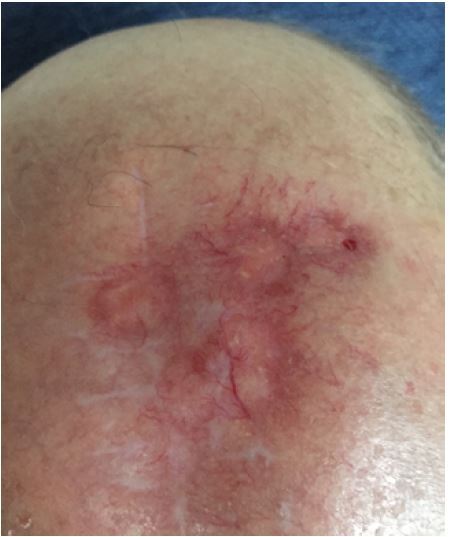
Post RT treatment for a CSCC.

RT is an overall safe procedure, although it can be associated with both acute and late toxicities. The most frequent acute toxicity may consist of acute, often erosive dermatitis, while late onset chronic depigmentation and telangiectasias are more often seen. Moreover, RT should not be recommended in younger (< 60 years old) patients because chronic toxicity becomes more visible with age. Higher doses per fraction lead to higher rates of late toxicity [27]. Therefore, accelerated fractionation schemes (acceleration means radiation treatment in which the total dose of radiation is given over a shorter period of time compared to standard radiation therapy) should be reserved for elderly and frail patients, or when the cosmetic outcome is less important. The volume to be irradiated in CSCCs represents the visible disease gross tumor volume (GTV) associated with microscopic disease and possible leakage pathways. The prescribed dose should therefore include all visible tumors plus an appropriate variable margin (clinical target volume), sparing surrounding healthy structures as much as possible [28,29]. Dosimetry and technical details should be monitored by a certified radiation oncologist, for some difficult anatomical sites there is the risk to undertreat some CSCC. RT may be combined with systemic therapies including chemotherapy (chemoradiation) or cetuximab in more advanced cases (for H/N tumours). Age is a very important issue for dose/fractionation decisions: as mentioned above, there are multiple dose fractionation schedules, but in patients < 50 years old RT fraction sizes of 2–2.5Gy are delivered over a period of 4–5 weeks, with the aim of achieving the best long-term results (heal and cosmetic outcome) [30]. When deciding on the number of fractions to prescribe for an appropriate course of radiotherapy, age must be considered together with the patient’s medical co-morbidity, performance status, and preference. In older (70–80 years old) patients it could be useful to decrease the total duration of treatment using daily RT fraction sizes of 3–4Gy over a period of 2–3 weeks (40–45Gy in 10–15 fractions). In elderly patients (>80 years old) less frequent (1 to 3 times per week) and larger fraction sizes are recommended, such as 5–7Gy in 5 to 6 fractions [31]. Hypofractionated RT delivered 2–3 times a week or once weekly is a highly effective option with tolerable treatment-related toxicity (Figures 1 and 2). Two recent systematic reviews of hypofractionated RT reported durable local control rates of over 90% and acceptable side effects [32]. In a systematic review comprising 40 relevant publications (external beam RT and brachytherapy included) of over 12 000 NMSC (24% SCC), local recurrence rates did not exceed 7.9%. The authors concluded that hypofractionated RT does not confer no obvious disadvantage in local control when compared with traditional more protracted RT schedules [33]. RT could have also an important role in palliative setting for bleeding tumors, to reduce disfiguring or symptomatic neoplasms: the most used schedules are 30 Gy in 10 daily fractions or 20 Gy in 5 fractions.

### Brachytherapy

Brachytherapy for cutaneous neoplasms has solid historical roots (with the first documented cases in 1896) [34]. Interest in the use of brachytherapy for skin cancers has decreased with the development of better surgical techniques such as Mohs surgery, and its application in skin cancer has declined significantly over the years. The introduction of the high-dose-rate afterloading technique and electronic brachytherapy has renewed interest in the role of brachytherapy in CSCC. Compared with external beam RT, high-dose-rate brachytherapy demonstrates some advantages, such as delivery of a high dose of radiation in the clinical target volume/planning target volume, rapid dose decrease at the periphery of the target, optimal sparing of normal tissue in sensitive structures, shorter treatment time, and use of a hypofractionated pathway. Cutaneous brachytherapy is advantageous especially in curved surfaces and should be considered instead of external beam radiation therapy (if surgical excision is not possible) in areas of poor vascularization, such as the back of the hands or feet or lower legs. It can be administered, for example, in a superficial technique using dermal applicators with 192iridium [35].

Interstitial brachytherapy is another option to deliver high dose radiation rate in thicker (above 5 mm) skin lesions with catheters to be inserted under anaesthesia directly into the lesion or surgical bed in the adjuvant setting [36]. Electronic brachytherapy is a new technique of RT based on a miniaturised X-ray source that allows to treat small and flat surfaced CSCC [37, 38] and has attracted considerable interest in recent years in the management of CSCC [39]. Although preliminary data on the use of electronic brachytherapy in CSCC are promising, there is a lack of scientific work designed for direct comparison with external beam RT or radionuclide brachytherapy. Because this is a relatively new scientific scenario, long-term follow-up data are also missing. The American Brachytherapy Society consensus statement does not endorse the use of electronic brachytherapy outside of prospective clinical studies. [40].

### RT Planning

**Figure 3:**
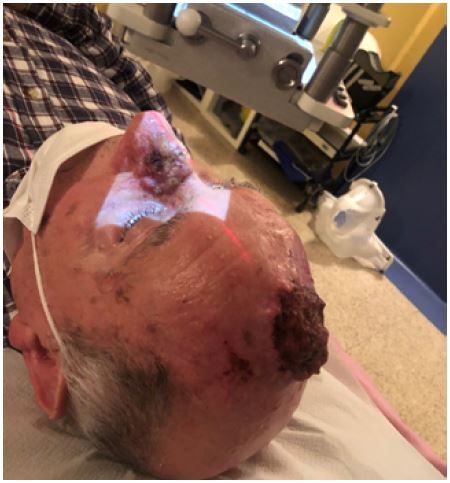
Treatment electron planning for a CSCC.

**Figure 4:**
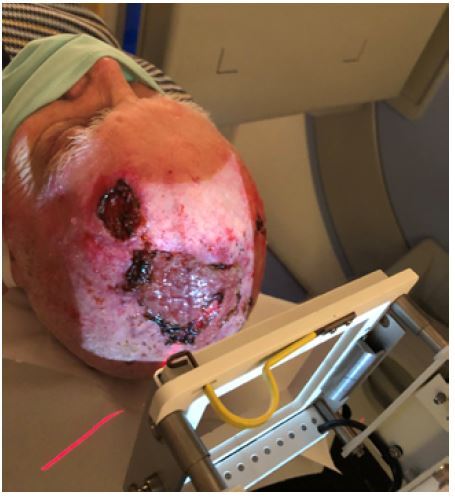
Treatment electron planning for a CSCC.

The main treatment planning modality in a modern radiation therapy department is based on computed tomography. Computed tomography is used to define the clinical target and organs at risk in photon and electron treatment (Figures 3 and 4). Integration with MRI imaging (for increased reliability for soft tissue) or PET (for biological assessment of disease) is often necessary. Radiation therapy results depend on accurate coverage of a target volume with appropriate margins. Margins that are too narrow can lead to local failure and margins that are too wide can increase radiation therapy-related morbidity. Delineating a target volume could be challenging, particularly in superficial and small CSCC, where the spatial resolution of computed tomography limits the visualization of any skin lesions. Multidisciplinary evaluation with the dermatologist (dermoscopic imaging) or surgeon (for surgical scar margins) is essential for the assessment of such targets [41]. In skin RT, the use of computed tomography-based planning is also linked to cases of larger and deeply invading CSCC, nodal basin RT, skin brachytherapy planning and in select palliative settings. After the clinical target and organs at risk delineation, a personalized plan is built up by the certified radiation oncologist in synergy with the medical physicist, respecting the dose limits of any organ at risk to minimize the side effects of radiation treatment.

## Conclusions

Radiotherapy could be an optimal therapeutic option for CSCC. It could be used for non-surgical candidates as exclusive therapy, in adjuvant high risk patients or to decrease pain and bleeding. Several dose schedules and techniques have been proposed both for EBRT (External beam radiotherapy) than for BRT (Brachytherapy). Multidisciplinary assessment is a main issue in this subset of patients.
